# Impact of HPV Testing Based on the 2020 Update of the German Cervical Cancer Screening Program—Data from a Retrospective Monocentric Study

**DOI:** 10.3390/cancers17122024

**Published:** 2025-06-17

**Authors:** Leonard Jung, Gilbert Georg Klamminger, Meletios P. Nigdelis, Elke Eltze

**Affiliations:** 1 Institute of Pathology, Saarbrücken-Rastpfuhl, 66113 Saarbrücken, Germany; 2 Department of General and Special Pathology, Saarland University (USAAR), Saarland University Medical Center (UKS), 66421 Homburg, Germany; 3 Department of Obstetrics and Gynecology, University Medical Center of the Johannes Gutenberg University Mainz, 55131 Mainz, Germany; 4 Department of Gynecology, Obstetrics and Reproductive Medicine, Saarland University Medical Center (UKS), 66424 Homburg, Germany

**Keywords:** cervical cancer, screening, detection rate, Germany, HPV-test, Pap-test, cytology, Co-test, combined screening

## Abstract

**Background/Objectives:** Since 2020, Germany has replaced its annual cytological screening for cervical cancer in women aged 35 and older with a combination screening program. This updated protocol, conducted every three years, includes both a cervical Pap smear and an HPV (human papillomavirus) test. In addition, the 2020 update of the German Cervical Cancer Screening Program mandates a more timely follow-up for certain abnormal findings compared to the previous approach. **Methods**: Results of cytologic, colposcopic, and histologic examinations between 2018 and 2021 were retrospectively retrieved from the electronic patient records of the Institute of Pathology Saarbrücken Rastpfuhl in order to present relevant findings within the years 2018–2019 and 2020–2021. The present study included all women who received appropriate follow-up based on the corresponding underlying screening modality. **Results**: Our reporting considered data from 30,715 women in 2018/19 (prior screening modality group) and 25,924 women in 2020/21 (updated screening modality group). In 2018/19, a total of 93 histological examinations were performed, compared to 237 in 2020/21. In 2018/19, 52 cases of CIN III and 12 cases of CIN II were identified, while in 2020/21, 78 cases of CIN III and 31 cases of CIN II were detected. In contrast, while women with negative cytological findings in 2018/19 were typically not referred for further colposcopic or histological evaluation, the updated program enabled earlier detection and treatment of cases with diagnosed high-grade dysplasia based solely on a positive HPV test. Notably, 31 women who were diagnosed at an earlier stage based solely on a positive HPV test initially presented with a cytological Pap I result (negative for intraepithelial lesion or malignancy; NILM). **Conclusions**: Additional HPV co-testing within the updated German cervical cancer screening program resulted not only in high rates of high-grade dysplasia detection but also a rise in the number of colposcopic procedures. While negative follow-up HPV findings regularly showed remission of the original finding, incidence of high-grade dysplasia was usually linked to a positive HPV test.

## Simple Summary

In 2020, Germany transitioned from annual cytological screening to a combination screening for cervical cancer in women aged 35 and older. The updated Cervical Cancer Screening Program, now conducted every three years, includes both a Pap smear and an HPV test. To evaluate the impact of this new protocol on the detection of high-grade cervical dysplasia (CIN II/III) and the frequency of colposcopic and histological examinations, we conducted a retrospective, single-center study. We present relevant screening outcomes from women examined between 2018–2019 and 2020–2021. Consistent with previous international studies, our findings show a higher number of high-grade dysplasia as well as a higher number of colposcopic procedures following the implementation of the new screening program.

## 1. Introduction

Since the introduction of the German cervical cancer screening program in 1971, the incidence of cervical carcinoma has decreased by almost 70%, a success largely attributed to the implementation of a cytology-based screening program [1,2]. However, HPV testing did not begin to play a significant role until the late 1990s. Initially used primarily as a triage tool, it is now employed as a primary screening method in national cervical cancer screening programs across several European countries, the United States, and Australia. This development was driven not only by the high prevalence of high-risk HPV in 99.7% of all cervical cancers [3], but also by data from large studies such as Swedescreen (Sweden), POBASCAM (Netherlands), ARTISTIC (England), and NTCC (Italy), which evaluated HPV testing with cytology as a primary screening tool and demonstrated results consistently endorsing the establishment of an HPV-based screening program [4,5,6,7]. Building on these findings, Ronco et al. conducted a mean follow-up of 6.5 years among participants from the aforementioned studies, concluding that HPV-based screening offers 60–70% greater protection against invasive cervical carcinoma than a cytology-based program [8]. Additional evidence such as the ATHENA study and the HPV FOCAL study further suggested the superiority of HPV testing in detecting high-grade CIN lesions [9,10]. The 2015 European Guidelines for Quality Assurance in Cervical Cancer Screening evaluated a comprehensive analysis of studies comparing primary HPV testing to cytology-based screening approaches. When considering ASC-US as the lowest cytological cut-off, HPV testing demonstrated a 37% (95% CI: 22–54%) higher sensitivity in comparison to cytology for detecting CIN II+ (i.e., cervical intraepithelial neoplasia grade II or worse) and a 43% (95% CI: 15–77%) higher sensitivity for detecting CIN III+ (i.e., cervical intraepithelial neoplasia grade III or worse) based on data from 11 European and North American screening studies [11]. Even when applying a stricter cytological threshold (≥LSIL), the relative sensitivity of HPV testing remained higher compared to cytology [11].

Although HPV testing showed lower specificity than cytology—with a relative specificity of 0.97 (95% CI: 0.96–0.98) using “Atypical Squamous Cells of Undetermined Significance” (ASC-US) as the cut-off and 0.92 (95% CI: 0.90–0.94) with (low grade squamous intraepithelial neoplasia) LSIL as the threshold [12]—it was found that incorporating appropriate triage strategies could improve diagnostic accuracy. For instance, using cytology as a triage following a positive HPV test resulted in a higher relative positive predictive value (PPV) for CIN II+, with a PPV ratio of 1.34 (95% CI: 1.04–1.72) compared to cytology alone [13].

Based on this large body of literature, Germany included HPV testing in its national cervical cancer screening program in 2020, although plans went back even further. Already in 2010, the Institute for Quality and Efficiency in Health Care (IQWiG) was commissioned to evaluate the benefits of employment of HPV testing as an initial screening tool. The 2010 IQWiG report already recognized the advantages of HPV-based or combined screening over a cytology-only approach (Final Report S10-01) [14]—a position further supported by a so called “Rapid Report” in 2014 (S13-03) [15] and by the 2015 supplements to the 2007 European guidelines for cervical cancer screening, both favoring the HPV-test as a primary testing method [16]. In January 2020, following this body of evidence, HPV testing was formally incorporated into Germany’s organized cervical cancer screening program. Although women between 20 and 34 receive annual pap smear testing, a combined test (HPV test and Pap test cytology) for women aged ≥35 is the primary new component of this program [17].

The aim of this retrospective single-center evaluation is to present pathological results from the periods 2018–2019 and 2020–2021. Particular focus is given to the impact of routine HPV testing in patients with negative or inconclusive Pap smear results.

## 2. Materials and Methods

Pathological data (including 56,639 Pap smear results) from the nationwide cervical cancer screening program between 2018 and 2021 were retrieved from electronic patient records in accordance with Annex 2 of the national QA agreement on cervical cytology (“Kassenärztliche Bundesvereinigung”, Berlin, Germany) at a single center (Institute of Pathology Saarbrücken Rastpfuhl, Saarbrücken, Germany). To also examine potential progression in cases of initially negative or low-risk cytology (Pap I/negative for intraepithelial lesion or malignancy; NILM) in combination with a positive HPV test, these cases were individually followed until 1 August 2023. Cytological findings were classified according to the 2014 Munich III Nomenclature (corresponding Bethesda equivalents are listed in Table S1), all histological results were classified according to clinical routine reporting in Germany (cervical intraepithelial neoplasia grade I/II/III; CIN). All patients screened according to the revised 2020 German cervical cancer screening guidelines and who completed appropriate follow-up were included in the study (inclusion criteria). Exclusion criteria encompassed women whose initial screening did not meet program requirements and those who did not participate in follow-ups. For detailed inclusion and exclusion criteria please refer to Table S2.

This study was authorized by the Medical Association of Saarland’s Ethics Committee (No. 143/23); the study adhered to the principles of the Declaration of Helsinki. All women were informed about the processing of their personal data and its use for research purposes through an invitation letter issued by their statutory health insurance providers. Women were granted the right to withdraw through a central opt-out office.

In this study, HPV positivity was defined as virological evidence of high-risk HPV infection (HPV type 16/18/31/33/35/39/45/51/52/56/58/59/66/68). Descriptive data presentation and processing was performed in accordance with a previous study led by Xhaja et al. [18]. All reported percentages were calculated using pivot tables and corresponding internal functions in Microsoft Excel (Version 16.86), with data rounded to two decimal places. Unless otherwise specified, percentages refer to the total number of women included in the screening program (2018/19: n = 30,715; 2020/21: n = 25,924).

## 3. Results

### 3.1. General Patient Data

In 2018, a total of 14,536 women participated in the cervical cancer screening program, and 16,179 women were screened in 2019. In 2020, clinico-cytological data—including age group, Pap smear result, HPV test results, and histological evaluations (if applicable)—were analyzed for 13,961 women, and for 11,963 women in 2021. The age distribution of participants across all study years ranged from 20 to 99 years. In 2020 and 2021, a higher proportion of younger women (20–34 years) participated, representing 34% of the total screened population compared to 25% in 2018/19. In contrast, in 2018 and 2019, the largest age group among screening participants was 50–64 years, comprising 32% of the 30,715 women. In total the median age was 52 (IQR = 36–64) within the screening period of 2018/19 and 43 (IQR = 30–57) within the screening period of 2020/2021. A decline in participation rate was observed for women aged 65 and older across all years from 2018 to 2021 (see Figure S1 for an exemplary visualization of the age structure in 2020).

The distributions of Pap groups and corresponding histological findings are shown in Supplementary Tables S3 and S4. In 2018 and 2019, cytologically inconspicuous results (Pap I/NILM) did not routinely undergo colposcopic follow-up. Similarly, findings with limited protective value (Pap Groups IIp/ASC-US, IIg/Atypical glandular endocervical cells not otherwise specified (AGC endocervical NOS), and IIe/Endometrial cells) did not routinely receive colposcopic evaluation according to national standards. Instead, HPV testing was performed six months later as a triage method; if positive, colposcopy was scheduled after an additional three months. During 2018 and 2019, a total of 93 histological evaluations were conducted (0.3% of 30,715 women). In contrast, in 2020 and 2021, cytologically negative findings and low-significance results accompanied by a positive HPV test were followed up according to the updated protocol. Altogether, the revised screening algorithm led to 237 histological examinations in 2020 and 2021, representing 0.91% of the 25,924 women screened.

### 3.2. Clinical Implications of HPV Testing and Cytology: Histological Progression, Repeated Testing, HPV Clearance, and Treatment Following Persistent Positivity

**Figure 1:**
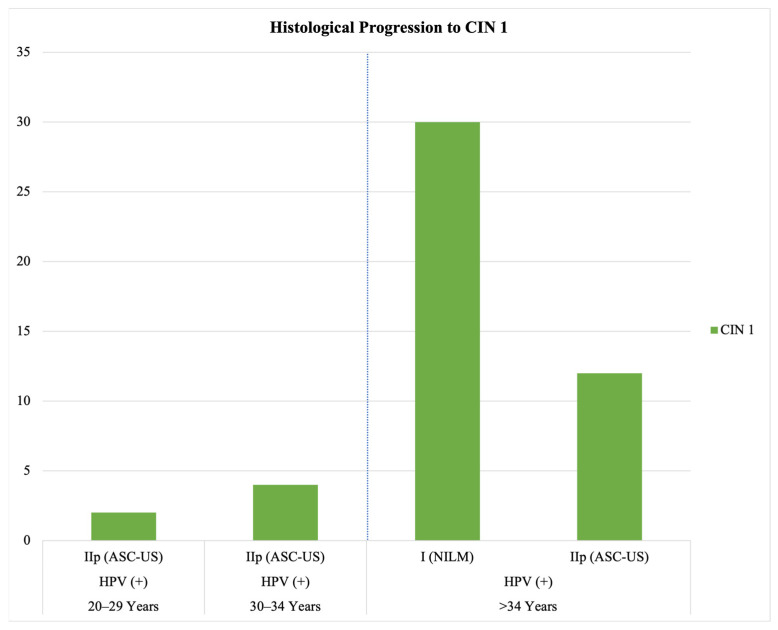
Progression from initial Pap group classification to CIN I in the years 2020/21 (display of a subset of data). All included cases tested positive for HPV. The associated age groups of affected women are shown.

Because systematic HPV testing was not in place in 2018 and 2019, histological progression could not be evaluated in cytologically negative but (truly) HPV-positive cases. During the 2020 screening period, 9167 women received an initial HPV test; 8524 (93%) tested negative, and 643 (7%) tested positive. Under the combined screening approach in 2020/21, a total of 30 women (0.12% of 25,924) with initially negative cytology (Pap I/NILM) progressed to CIN I during follow-up. All of these women were over 34 years of age and had tested positive for HPV (see Figure 1).

**Figure 2:**
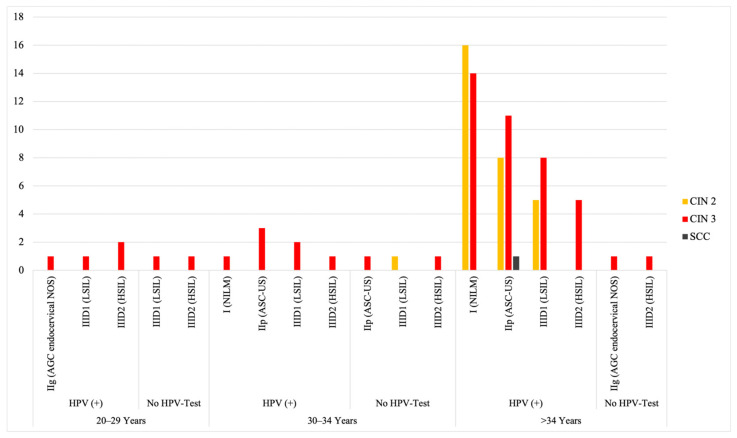
Histological progression in relation to initial cytological results (display of a subset of data), HPV status, and age group (*X*-axis). The *Y*-axis indicates the number of women. Notably, more women with initially negative cytology progressed to CIN II+ than those with any other initial cytological category. Data reflect follow-up outcomes from the 2020 screening cohort.

Additionally, 16 women (0.06% of 25,924) with initially negative cytology (Pap I/NILM) but persistent HPV positivity developed CIN II on histological follow-up; all were over 34 years of age. Fifteen women with initially negative cytology and a positive HPV test progressed to CIN III: 1 woman (0.004% of 25,924) belonged to the 30–34 age group, while the remaining 14 (0.05% of 25,924) were over 34 years old. As summarized in Figure 2 (as well as Table S5 and Figure S2), a total of 31 women with initially negative cytology (Pap I/NILM) were diagnosed with high-grade dysplasia solely on the basis of HPV positivity, regardless of age group.

Among 199 women (0.77% of 25,924) with initially negative cytology (Pap I/NILM) and high-risk HPV infection, spontaneous HPV clearance was observed at the 12-month follow-up test without any clinical intervention (see Figure S3). This clearance occurred exclusively in women with initial Pap classifications of I (NILM), IIa (NILM), IIp (ASC-US), or IIID1 (LSIL).

Following biopsy without evidence of dysplasia, 27 women (0.11% of 25,924) with initially negative cytology showed HPV clearance. Of these, 21 underwent colposcopy and biopsy due to persistent HPV infection at 12 months, in accordance with the follow-up algorithm. In six cases, persistence of infection was accompanied by progression to Pap IIp (ASC-US), prompting further colposcopic and histological evaluation. Ultimately, in all 27 women, HPV infection was no longer detectable in subsequent follow-up tests, and cytological results remained negative (see Figure S4).

During the 2020 screening, 31 women (0,22% of 13,961) developed high-grade dysplasia despite initial negative cytology. This group includes two women (0.01%) who underwent colposcopic evaluation at first follow-up, contrary to protocol; histological analysis confirmed CIN II in both cases. Of the 31 women who developed high-grade dysplasia following an initial Pap I (NILM) result, 15 (0.11%) demonstrated repeat negative cytology. Subsequent colposcopy and histology revealed CIN II in seven (0.05%) and CIN III in eight women (0.06%).

Six women (0.04%) progressed from Pap I (NILM) to Pap IIp (ASC-US), and one (0.01%) to Pap IIg (AGC endocervical NOS). Among these, five were confirmed HPV-positive on retesting. Histology confirmed CIN II in four of these seven cases (0.03%) and CIN III in the remaining three (0.02%).

Similarly, three women (0.02%) with persistent HPV infection and Pap IIID1 (LSIL) at follow-up were diagnosed with CIN II in two cases (0.01%) and CIN III in one case (0.01%). In addition, four women (0.03%) presented with higher-grade cytological abnormalities during follow-up: one with Pap IIID2 (high-grade squamous intraepithelial lesion [HSIL]) and three with Pap IVa-p (HSIL). All three Pap IVa-p (0.02%) cases as well as the women with Pap IIID2 (0,01%) were confirmed as CIN III on histology. 

Figures S2 and S5 provide a visual summary of HPV-related dysplasia detection in relation to concurrent cytological findings.

## 4. Discussion

Persistent HPV infection is recognized as a necessary condition for the development of cervical cancer [3,19,20]; in fact, the absence of HPV virtually excludes the possibility of cervical carcinogenesis [21]. This makes HPV infection a critical target for prevention strategies and highlights the value of HPV testing in reducing the future incidence of cervical neoplasms. The importance of this approach is underscored by global scientific recommendations advocating the routine use of HPV testing in cervical cancer screening, as well as by the World Health Organization’s (WHO) commitment to eliminating cervical cancer as a public health problem [22].

The present study aimed to evaluate the clinical relevance of HPV testing during the first year of the newly implemented German cervical cancer screening program, with particular emphasis on real-world outcomes in women with negative cytology but positive HPV test results. The updated screening guidelines mark a major advancement as they incorporate routine HPV testing alongside cytological screening: women aged 35 and older now undergo combined HPV and Pap testing every three years.

The former German national guideline on cervical cancer prevention (December 2017) recommended HPV testing for women over the age of 29 if Pap group classifications IIp (ASC-US), IIg (AGC endocervical NOS), or IIID1 (LSIL) persisted for six months [23]. In cases where HPV infection was confirmed, colposcopic follow-up was advised within three months. This approach remains unchanged in the current screening program for women under 35. For women aged 35 and older; however, the updated guideline recommends colposcopic evaluation within three months for all cases of Pap IIp (ASC-US), IIg (AGC endocervical NOS), or IIID1 (LSIL), if accompanied by a positive HPV test result. This change effectively shortens the follow-up interval by up to six months compared to the previous protocol. A similar acceleration applies to initially negative cytological results—Pap I or IIa (both NILM)—when combined with a positive HPV test. In such cases, follow-up is triggered by either cytological progression to at least Pap group IIp (ASC-US), IIg (AGC endocervical NOS), or IIe (endometrial cells), or by a repeated positive HPV result, regardless of cytology. Under the updated protocol, follow-up may thus occur at least 12 months earlier than under the previous approach for HPV-positive Pap I (NILM) findings.

In 2018 and 2019, dysplastic changes or histological progression in women with negative cytology but undetected HPV infection would likely have remained unnoticed until later annual follow-up visits—assuming such visits were consistently attended. In Germany, screening participation among women under 55 is estimated at 70–80%, compared to the broader European average of approximately 65.4% [24,25].

Our study shows that the introduction of routine HPV testing led to an increased detection of high-grade squamous intraepithelial lesions and enabled earlier intervention: 31 women with initially negative cytology (Pap I [NILM]) were diagnosed and treated earlier since 2020. This indicates that HPV testing has significantly improved surveillance in cytologically inconspicuous cases. That said, the population of women aged 35 and older eligible for cervical cancer screening in Germany currently stands at approximately 29.9 million [26]. Based on our findings, an estimated benefit for over 115,000 women nationwide may be assumed as a result of the updated screening protocol.

Furthermore, we also observed an increase in the number of colposcopies performed, along with a rise in detected dysplastic lesions following the implementation of the updated screening program. Specifically, colposcopies increased from 93 cases in 2018/2019 to 237 cases in 2020/2021; a trend also recently reported by the readable study of Xhaja et al. [18]. While the number of high-grade lesions consistent with CIN III remained relatively stable, it still rose numerically from 52 to 78 cases. In contrast, detection rates of CIN II and CIN I increased substantially by the end of the study period, from a combined 14 cases in 2018/2019 to 73 in 2020/2021. All CIN I–III cases were associated with a positive HPV test, underscoring the high sensitivity of HPV testing for detecting cervical dysplasia. This finding is consistent with previous research, such as the study by Cuzick et al. (2006), which reported sensitivity rates of 96.1% for HPV testing versus 53% for cytology alone [27].

Conversely, the increased sensitivity of HPV testing may lead to a higher number of false-positive results, potentially resulting in unnecessary colposcopic interventions and their associated side effects, such as pain, bleeding, and discharge—symptoms reported by 82% of women following the procedure [28]. In our study, spontaneous HPV clearance was observed in 224 women (regardless of initial Pap classification; see Supplementary Figure S3), with the highest clearance rate (n = 199) occurring in women with Pap I (NILM) cytology combined with a positive HPV test. Concerns about overtreatment and the potential strain on healthcare systems caused by the high sensitivity of HPV testing have been addressed in studies such as the HPV FOCAL trial and Swedescreen. These studies showed that while HPV-based screening initially leads to higher colposcopy rates, these rates tend to decline over time as programs mature—suggesting early detection rather than overuse [5,10]. Moreover, the implementation of HPV testing systems with improved specificity could further mitigate overtreatment. For instance, recent data showed that the Hybrid Capture 2 HPV test (Qiagen, Venlo, Netherlands) led to significantly more colposcopic referrals compared to the Aptima HPV test (Hologic, Marlborough, MA, USA) (60.8 vs. 38.3 per 1000 women; *p* < 0.001), although no significant differences were found between the two systems in detecting CIN II–III lesions [29]. Importantly, as HPV vaccination coverage increases, a decline in HPV prevalence is expected, which will likely reduce the overall number of colposcopic procedures required [30]. Simultaneously, shifts in HPV type distribution may lead to a relative increase in low-grade dysplasia caused by less oncogenic HPV strains not yet covered by current vaccines [31,32].

These findings support the notion that a primary HPV testing strategy—with cytology used as a triage tool—may be appropriate in the future. In our cohort, no cases of high-grade squamous intraepithelial lesions or Pap categories ≥ Pap IIID2 (HSIL) were observed in HPV-negative women. Additionally, cytological abnormalities such as Pap IIID1 (LSIL) and low-risk categories including Pap IIp (ASC-US), IIg (AGC endocervical NOS), and IIe (endometrial cells) showed reliable regression when combined with a negative HPV result.

However certain limitations of this study such as the retrospective design, the monocentric setting, as well as the lack of longer follow-up periods need to be considered when interpreting our proposed findings. Additionally, due to method of data collection—setting a focus on the cases from a pathological perspective and therefore screening the pathological results/reports—we were not able to retrieve more detailed clinical information of the individual patients. Furthermore vaginal smears following hysterectomy were not included in the analysis. Last but not least our study did not aim at direct comparison of sensitivity/specificity—the employed method of data presentation was defined in comparison to a previous observational study on cervical cancers screening programs in Germany [18].

From a socioeconomic perspective, cytological screening remains a labor-intensive and resource-consuming method. It requires highly trained personnel, generates substantial material costs, and is prone to interpretive variability due to factors such as blood, mucus, poor fixation, and uneven cell distribution. These limitations contribute to the wide range of reported sensitivities (30–87%) [33,34,35,36]. While shortening screening intervals could theoretically improve cytology performance, this would significantly diminish the cost-effectiveness of the approach [37].

In contrast, HPV testing offers high reproducibility, less observer variability, fewer training demands for clinicians, and greater throughput capacity [38,39,40]. Thus, concerns about the financial, technical, and logistical burden of implementing HPV-based primary screening in Germany appear unwarranted. On the contrary, the evidence supports the long-term potential of HPV testing to reduce both healthcare costs and disease burden.

## 5. Conclusions

This retrospective monocentric study evaluated the impact of the updated German cervical cancer screening program—introduced in 2020, which includes HPV co-testing in addition to Pap cytology—for women aged 35 and older. Pathological data from over 56,000 women screened between 2018 and 2021 were presented. We compared screening results as well as follow-up outcomes between the old cytology-only protocol (2018–2019) and the updated combination screening (2020–2021), focusing particularly on the prognostic relevance of HPV positivity in cytologically inconspicuous cases. That said, our study highlights several key findings that may serve as a basis for hypothesis generation in future research.

- Since we report a rise in CIN II/III diagnoses in 2020/21 compared to 2018/19, the implementation of HPV co-testing will increase the detection rate of high-grade cervical dysplasia.
- No histological progression was observed in HPV-negative cases with initial abnormal cytology within our study—a negative HPV test correlates with a low risk of progression and potential spontaneous regression.
- As already determined in studies such as Swedescreen, Pobascam, Artistic, and HPV-Focal trial, an initially higher number of colposcopies is observed [4,5,7,10]. In our study, the overall number of colposcopic interventions increased due to the updated screening program. However, it remains to be resolved whether these referral rates can be considered as an earlier detection of dysplastic lesions rather than overdiagnosis per se (as postulated in the abovementioned studies).

The updated cervical cancer screening protocol incorporating HPV co-testing offers clear advantages: it improves detection of high-grade cervical intraepithelial neoplasia (CIN II/III), particularly in HPV-positive women with negative cytology, enabling earlier intervention. Compared to cytology, HPV testing is more sensitive, reproducible, and allows for extended screening intervals in HPV-negative individuals [8,9,10]. However, increased sensitivity may lead to more false positives, unnecessary colposcopies, and added burden on patients and healthcare systems. These limitations emphasize the importance of optimizing triage strategies to reduce overtreatment while maintaining the benefits of early detection. Future studies should evaluate the long-term effects of the updated screening program, particularly regarding trends in colposcopy rates over time. Research is also needed to assess the cost-effectiveness of different HPV testing platforms and to optimize follow-up protocols for HPV-positive, cytology-negative cases. Finally, increasing HPV vaccination coverage and its influence on HPV prevalence and dysplasia rates should be investigated as it may reduce the need for invasive diagnostic procedures and reshape future screening strategies.

## Data Availability

The original contributions presented in this study are included in the article/Supplementary Material. Further inquiries can be directed to the corresponding author.

## Supplementary Materials

The following supporting information can be downloaded at: https://www.mdpi.com/article/10.3390/cancers17122024/s1, Figure S1: Age Structure of Women at the beginning of Screening, Figure S2: Developmental steps until intraepithelial lesion for age group > 34 years, Figure S3: Elimination of HPV-Infection by host immune system, Figure S4: Elimination of HPV-Infection after biopsy, Figure S5: Earlier detection due to a positive HPV-test result, Table S1: Munich Nomenclature III and its correlate in the Bethesda System, Table S2: Summary of inclusion and exclusion criteria, Table S3: Annual cervical cytology statistics from 2018 to 2019, Table S4: Annual cervical cytology statistics from 2020 to 2021, Table S5: Histological progression in relation to the belonging age group, HPV-test result and the cytological outcome.

## Abbreviations

The following abbreviations are used in this manuscript:

HPV	Human Papillomavirus
CIN	Cervical intraepithelial neoplasia
IQWiG	Institute for Quality and Efficiency in Health Care
QA	Quality Assurance
WHO	World Health Organization
NILM	Negative for intraepithelial lesion or malignancy
ASC-US	Atypical squamous cells of undetermined significance
AGC endocervical NOS	Atypical glandular endocervical cells not otherwise specified
LSIL	Low-grade squamous intraepithelial lesion
HSIL	High-grade squamous intraepithelial lesion
AIS	Adenocarcinoma in situ
