# Supplementary material for: Impact of HPV Testing Based on the 2020 Update of the German Cervical Cancer Screening Program—Data from a Retrospective Monocentric Study
Source: Cancers (Basel). 2025 Jun 17;17(12):2024. doi: 10.3390/cancers17122024 (PMC12190846; doi:10.3390/cancers17122024)
Supplement: Supplementary file 1 [file cancers-17-02024-s001.zip › cancers-3600669-supplementary.pdf]

Supplementary Materials

Figure S1: Age structure of women at the beginning of screening

Figure S2: Developmental steps until intraepithelial lesion for age group > 34 years

Figure S3: Elimination of HPV-Infection by host immune system

Figure S4: Elimination of HPV-Infection after biopsy

Figure S5: Earlier detection due to a positive HPV-test result

Table S1: Munich Nomenclature III and its correlate in the Bethesda System.

Table S2: Summary of inclusion and exclusion criteria.

Table S3: Annual cervical cytology statistics from 2018 to 2019

Table S4: Annual cervical cytology statistics from 2020 to 2021

Table S5: Histological progression in relation to the belonging age group, HPV-test result and the cytological outcome.

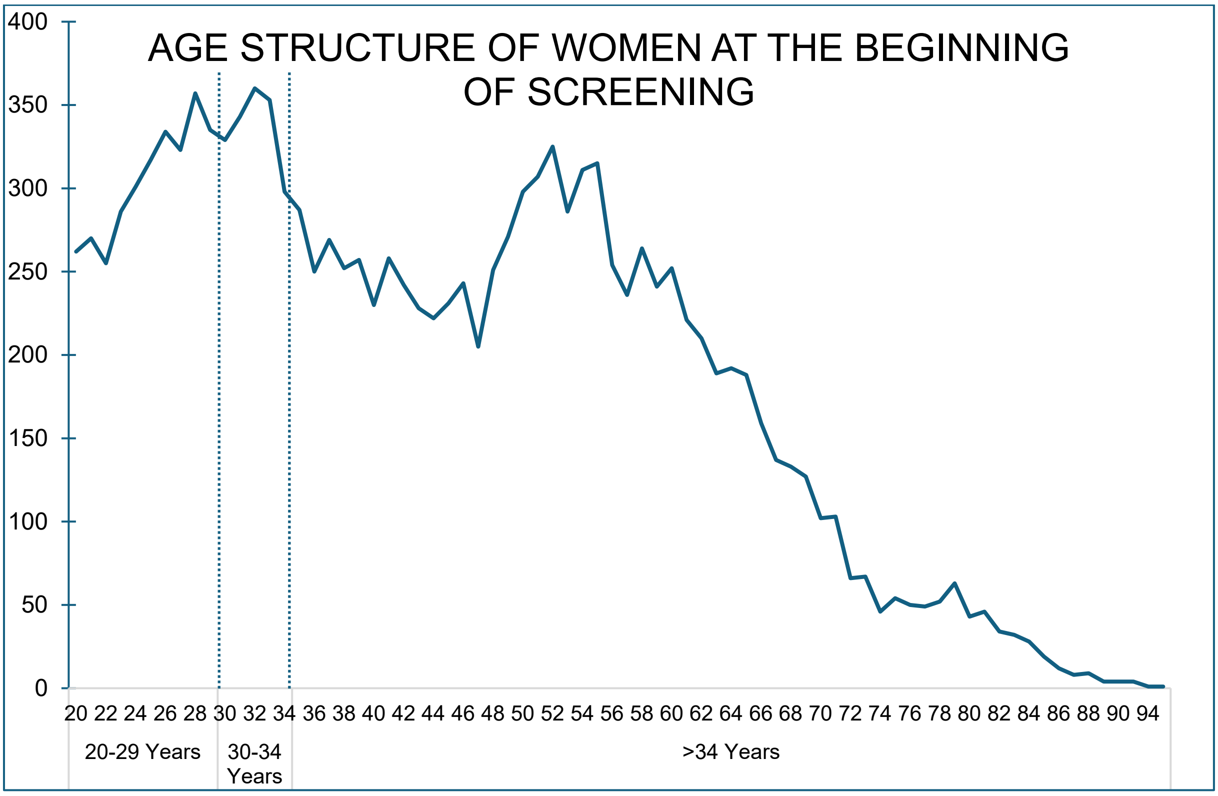

Figure S1. Depiction of womens' ages, who participated at the revised german cervical cancer screening program in 2020. The X-axis represents the corresponding age groups, the Y-axis indicates the number of women.

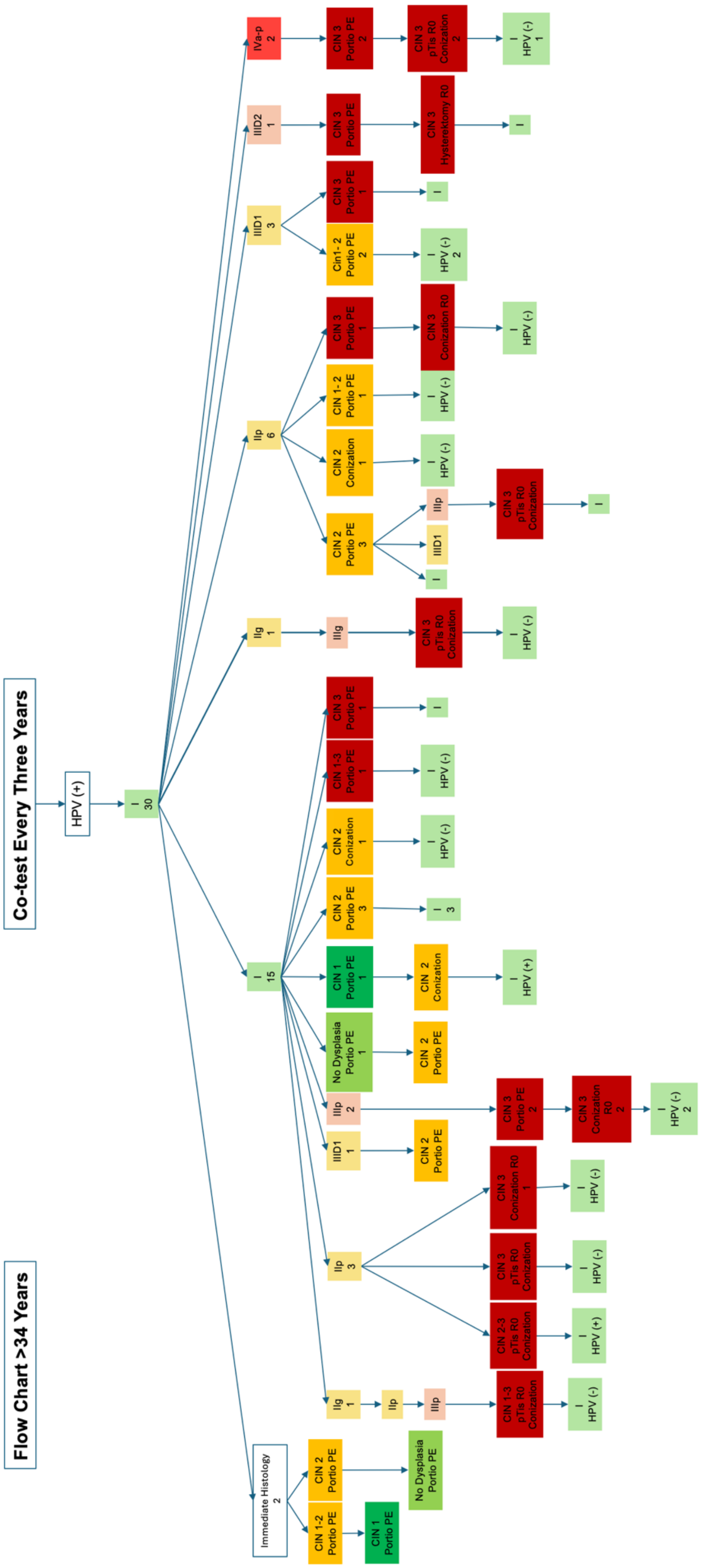

Figure S2. Flow chart for women aged over 34 years, illustrating the diagnostic pathway from initial negative cytology with a positive HPV test to the eventual detection of intraepithelial lesions. Two women underwent immediate excisional biopsy. Based on follow-up data from the 2020 screening cohort.

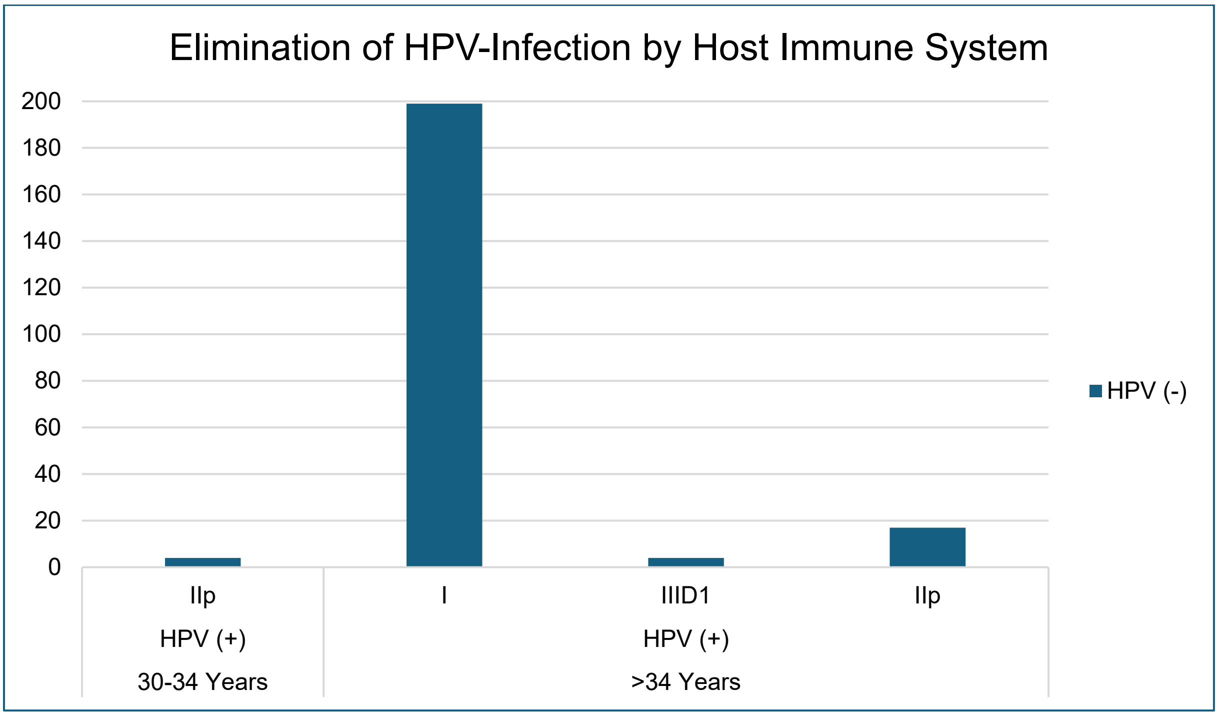

Figure S3. The horizontal axis displays age groups, initial HPV test results, and baseline cytological findings. The vertical axis indicates the number of women. As shown, spontaneous HPV clearance occurred in cases with NILM (Pap I/IIa), ASC-US (Pap IIp), or LSIL (Pap IIID1) cytology, consistent with effective immune response. Based on follow-up data from the 2020 screening cohort.

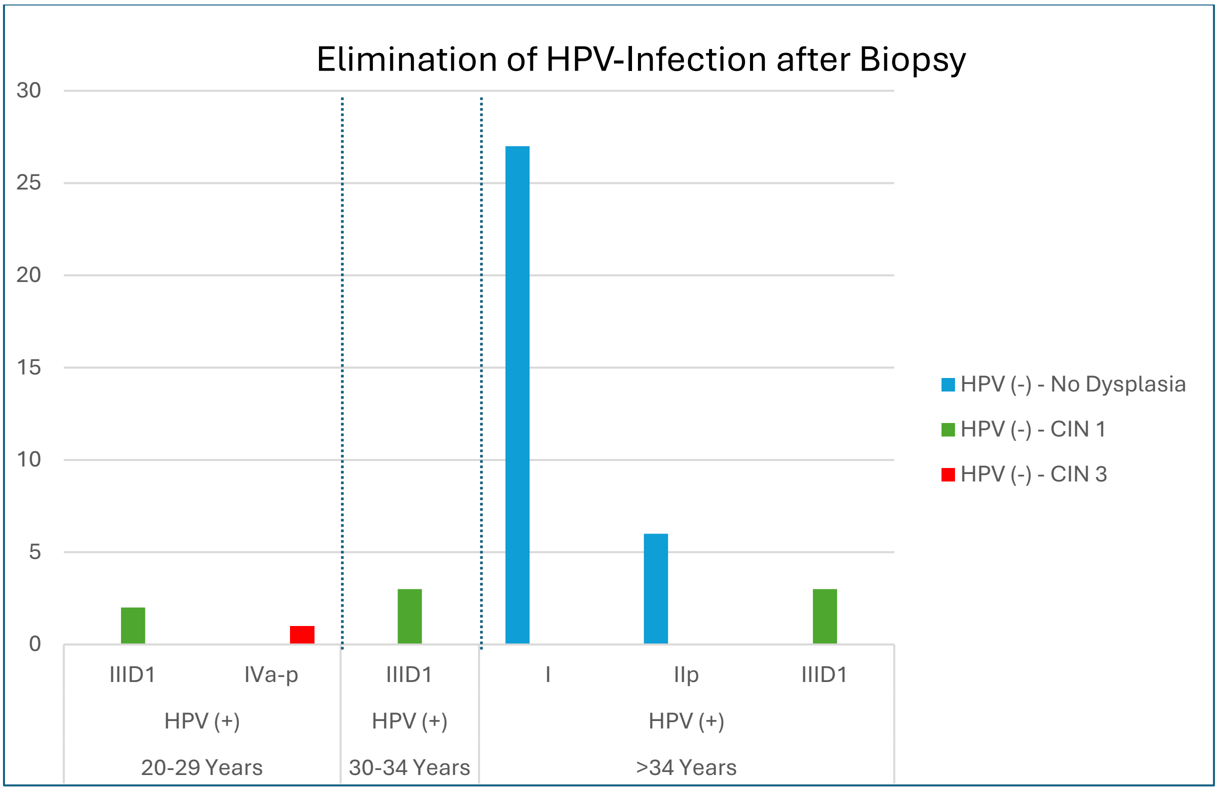

Figure S4. After persistence of viral infection for at least 12 months, a biopsy was taken which revealed no present dysplasia. In the combined testing after biopsy there was no detectable HPV-infection either. Interestingly younger women had higher Pap-results at the beginning that were not affiliated with the histological findings. Based on follow-up data from the 2020 screening cohort.

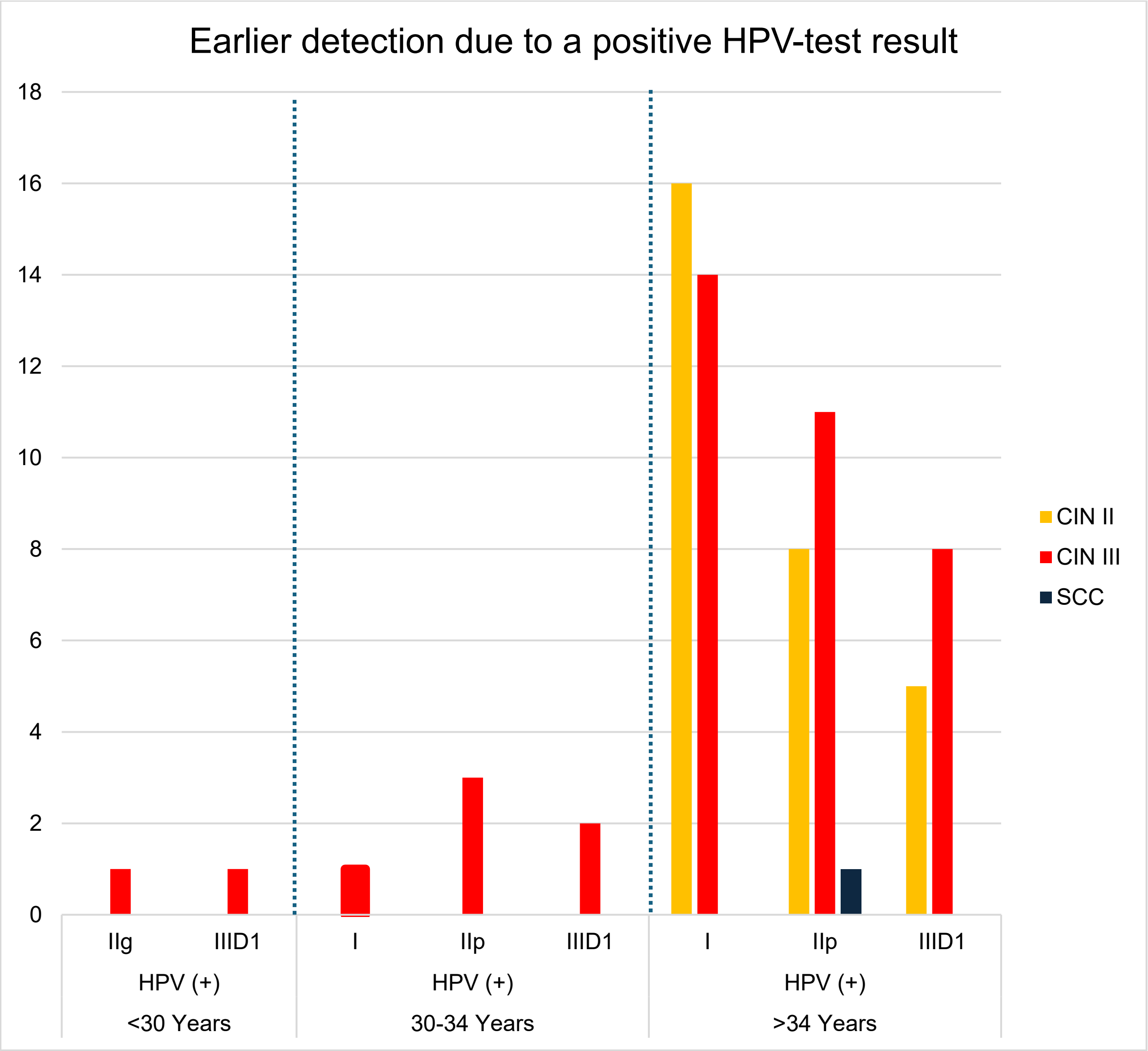

Figure S5. Distribution of initial Pap group, HPV test result, and age group (X-axis) in relation to the number of women diagnosed with high-grade squamous intraepithelial lesions (Y-axis). Owing to the revised screening algorithm, cases of HSIL associated with Pap IIp and IIID1, as well as one case of cervical squamous cell carcinoma, were identified approximately six months earlier than they would have been under the previous system. Based on follow-up data from the 2020 screening cohort.

Table S1: Munich Nomenclature III and its correlate in the Bethesda System.

Munich Nomenclature III	Bethesda System
0	Unsatisfactory for evaluation
I	NILM
IIa	NILM
IIp	ASC-US
IIg	AGC endocervical NOS
IIe	Endometrial cells
IIIp	ASC-H
IIIg	AGC endocervical favor neoplastic
IIIe	AGC endometrial
IIIx	AGC favor neoplastic
IIID1	LSIL
IIID2	HSIL
IVa-p	HSIL
IVa-g	AIS
IVb-p	HSIL with features suspicious for invasion
IVb-g	AIS with features suspicious for invasion
Vp	Squamous cell carcinoma
Vg	Endocervical adenocarcinoma
Ve	Endometrial adenocarcinoma
Vx	Other malignant neoplasms

Table S2. Summary of inclusion and exclusion criteria.

Inclusion Criteria	Exclusion Criteria
Age ≥ 20 years	Age < 20 years
Adapted Screening (combined testing age ≥ 35)	Non adapted Screening (no combined testing age ≥ 35)
Regular follow up (in case of necessary recheck)	No follow up (in case of necessary recheck)
No privately financed reexamination	Duplicate due to privately financed reexamination

Table S3. Annual cervical cytology statistics from 2018 to 2019, based on Annex 2 of the National Quality Assurance Agreement on Cervical Cytology. Malignancies unrelated to cervical squamous cell carcinoma are not included. Correspondences between the Munich III Nomenclature and the Bethesda System are as follows: Pap I/ IIa: NILM, Pap IIp: ASC-US, Pap IIg: AGC endocervical NOS, Pap IIe: Endometrial cells, Pap IIIp: ASC-suspicious for HSIL, Pap IIIg: AGC endocervical favor neoplastic, Pap IIIe: AGC endometrial, Pap IIIx: AGC favor neoplastic, Pap IIID1: LSIL, Pap IIID2/ IVa-p: HSIL, Pap IVa-g: AIS (Adenocarcinoma in situ), Pap IVb-p: HSIL with features suspicious for invasion, Pap IVb-g: AIS with features suspicious for invasion, Vp: Squamous cell carcinoma, Vg: Endocervical adenocarcinoma, Ve: Endometrial adenocarcinoma, Vx: Other malignant neoplasms. It should be noted that only 79 histological examinations are included, since adenocarcinomas, endometrial carcinomas, and other malignancies were not considered in this table.

Year	PAP	Negative		Group II			Group III				Group IIID		Group IV				Group V					
		I	IIa	IIp	IIg	IIe	IIIp	IIIg	IIIe	IIIx	IIID1	IIID2	IVa-p	IVa-g	IVb-p	IVb-g	V-p	V-g	V-e	V-x	0	Total
2018	Women	13971<br>96.11%	11<br>0.08%	333<br>2.29%	66<br>0.45%	43<br>0.3%	7<br>0.05%	5<br>0.03%	2<br>0.01%	2<br>0.01%	43<br>0.3%	22<br>0.15%	16<br>0.11%	1<br>0.01%	3<br>0.02%	1<br>0.01%	2<br>0.01%	1<br>0.01%	1<br>0.01%		6<br>0.04%	14536<br>100%
	No Hint for CA Precursor or CA						1<br>0.01%	1<br>0.01%	1<br>0.01%													3<br>0.02%
	CIN 1																					0
	CIN 2										2<br>0.01%	1<br>0.01%	1<br>0.01%	1<br>0.01%								5<br>0.03%
	CIN 3						1<br>0.01%	3<br>0.02%				5<br>0.03%	11<br>0.08%				1<br>0.01%					21<br>0.23%
	Cervical Squamous Cell Carcinoma												1<br>0.01%		2<br>0.01%		1<br>0.01%	1<br>0.01%				5<br>0.03%
2019	Women	15477<br>95.66%	8<br>0.05%	444<br>2.74%	57<br>0.35%	51<br>0.32%	9<br>0.06%	6<br>0.04%	4<br>0.02%		63<br>0.39%	22<br>0.14%	27<br>0.17%	2<br>0.01%	2<br>0.01%						7<br>0.04%	16179<br>100%
	No Hint for CA Precursor or CA							1<br>0.01%						1<br>0.01%								2<br>0.01%
	CIN 1										1<br>0.01%		1<br>0.01%									2<br>0.01%
	CIN 2						1<br>0.01%	1<br>0.01%			3<br>0.02%		2<br>0.01%									7<br>0.04%
	CIN 3						1<br>0.01%	1<br>0.01%			3<br>0.02%	4<br>0.02%	21<br>0.14%		1<br>0.01%							31<br>0.19%
	Cervical Squamous Cell Carcinoma												2<br>0.01%		1<br>0.01%							3<br>0.02%

Table S4. Annual cervical cytology statistics from 2020 to 2021, based on Annex 2 of the National Quality Assurance Agreement on Cervical Cytology. Malignancies unrelated to cervical squamous cell carcinoma are not included. The corresponding Bethesda System equivalents for the Munich III Nomenclature are as follows: Pap I/ IIa: NILM, Pap IIp: ASC-US, Pap IIg: AGC endocervical NOS, Pap IIe: Endometrial cells, Pap IIIp: ASC-suspicious for HSIL, Pap IIIg: AGC endocervical favor neoplastic, Pap IIIe: AGC endometrial, Pap IIIx: AGC favor neoplastic, Pap IIID1: LSIL, Pap IIID2/ IVa-p: HSIL, Pap IVa-g: AIS, Pap IVb-p: HSIL with features suspicious for invasion, Pap IVb-g: AIS with features suspicious for invasion, Vp: Squamous cell carcinoma, Vg: Endocervical adenocarcinoma, Ve: Endometrial adenocarcinoma, Vx: Other malignant neoplasms. It should be noted that only 223 histological examinations are included, since adenocarcinomas, endometrial carcinomas, and other malignancies were not considered in this table.

Year	PAP	Negative		Group II			Group III				Group IIID		Group IV				Group V					
		I	IIa	IIp	IIg	IIe	IIIp	IIIg	IIIe	IIIx	IIID1	IIID2	IVa-p	IVa-g	IVb-p	IVb-g	V-p	V-g	V-e	V-x	0	Total
2020	Women	13401 95.99%	6 0.04%	322 2.31%	18 0.13%	59 0.42%	11 0.08%	3 0.02%	3 0.02%		76 0.54%	21 0.15%	20 0.14%	1 0.01%	1 0.01%		4 0.03%			1 0.01%	14 0.1%	13961 100%
	No Hint for CA Precursor or CA			9 0.06%	1 0.01%		2 0.01%				4 0.03%	4 0.03%										20 0.14%
	CIN 1	1 0.01%		10 0.07%				1 0.01%			13 0.09%	2 0.01%										27 0.19%
	CIN 2			4 0.03%				1 0.01%			8 0.05%	2 0.01%	2 0.01%									17 0.11%
	CIN 3			5 0.04%			5 0.04%	1 0.01%			5 0.04%	8 0.05%	17 0.11%	1 0.01%								42 0.3%
	Cervical Squamous Cell Carcinoma																2 0.01%					2 0.01%
2021	Women	11505 96.17%	6 0.05%	265 2.22%	8 0.07%	29 0.24%	7 0.06%	3 0.03%	4 0.03%		77 0.64%	32 0.27%	16 0.13%	2 0.02%	1 0.01%		3 0.03%			1 0.01%	4 0.03%	11963 100%
	No Hint for CA Precursor or CA	27 0.23%		10 0.08%		1 0.01%					4 0.03%	4 0.03%										46 0.38%
	CIN 1	2 0.02%		2 0.02%	1 0.01%		1 0.01%	1 0.01%			6 0.05%	2 0.02%										15 0.14%
	CIN 2	2 0.02%		2 0.02%			1 0.01%	1 0.01%			4 0.03%	4 0.03%										14 0.12%
	CIN 3			1 0.01%			4 0.03%				9 0.08%	10 0.08%	11 0.09%	1 0.01%								36 0.3%
	Cervical Squamous Cell Carcinoma												3 0.03%		1 0.01%							4 0.03%

Table S5. Display of a subset of data (see also manuscript Figure 2): Histological progression in relation to the belonging age group, HPV-test result and the cytological outcome. Adapted to the follow up of 2020.

Age Group	HPV-Test	Pap-Group	CIN 2	CIN 3	SCC	Total
20-29 Years	HPV (+)	IIg		1		1 (1.18%)
		IIID1		1		1 (1.18%)
		IIID2		2		2 (2.35%)
	No HPV-Test	IIID1		1		1 (1.18%)
		IIID2		1		1 (1.18%)
30-34 Years	HPV (+)	I		1		1 (1.18%)
		IIp		3		3 (3.53%)
		IIID1		2		2 (2.35%)
		IIID2		1		1 (1.18%)
	No HPV-Test	IIp		1		1 (1.18%)
		IIID1	1			1 (1.18%)
		IIID2		1		1 (1.18%)
>34 Years	HPV (+)	I	16	14		30 (34.12%)
		IIp	8	11	1	20 (23.53%)
		IIID1	5	8		13 (15.29%)
		IIID2		5		5 (5.88%)
	No HPV-Test	IIg		1		1 (1.18%)
		IIID2		1		1 (1.18%)
**Total**			30 (35.29%)	55 (63.53%)	1 (1.18%)	86 (100%)
